# Cardiorespiratory and Thermoregulatory Parameters Are Good Surrogates for Measuring Physical Fatigue during a Simulated Construction Task

**DOI:** 10.3390/ijerph17155418

**Published:** 2020-07-28

**Authors:** Shahnawaz Anwer, Heng Li, Maxwell Fordjour Antwi-Afari, Waleed Umer, Arnold Y. L. Wong

**Affiliations:** 1 Department of Building and Real Estate, Hong Kong Polytechnic University, Hong Kong SAR, China; heng.li@polyu.edu.hk (H.L.); maxwell.antwiafari@connect.polyu.hk (M.F.A.-A.); 2 Department of Construction Engineering and Management, King Fahd University of Petroleum & Minerals, Dhahran 31261, Saudi Arabia; Waleed.umer@kfupm.edu.sa; 3 Department of Rehabilitation Sciences, Hong Kong Polytechnic University, Hong Kong SAR, China; arnold.wong@polyu.edu.hk

**Keywords:** physiological measures, heart rate, skin temperature, construction workers, health and safety

## Abstract

Cardiorespiratory (e.g., heart rate and breathing rate) and thermoregulatory (e.g., local skin temperature and electrodermal activity) responses are controlled by the sympathetic nervous system. To cope with increased physical workload, the sympathetic system upregulates its activity to generate greater sympathetic responses (i.e., increased heart rate and respiratory rate). Therefore, physiological measures may have the potential to evaluate changes in physical condition (including fatigue) during functional tasks. This study aimed to quantify physical fatigue using wearable cardiorespiratory and thermoregulatory sensors during a simulated construction task. Twenty-five healthy individuals (mean age, 31.8 ± 1.8 years) were recruited. Participants were instructed to perform 30 min of a simulated manual material handling task in a laboratory. The experimental setup comprised a station A, a 10-metre walking platform, and a station B. Each participant was asked to pick up a 15 kg ergonomically-designed wooden box from station A and then carried it along the platform and dropped it at station B. The task was repeated from B to A and then A to B until the participants perceived a fatigue level > 15 out of 20 on the Borg-20 scale. Heart rate, breathing rate, local skin temperature, and electrodermal activity at the wrist were measured by wearable sensors and the perceived physical fatigue was assessed using the Borg-20 scale at baseline, 15 min, and 30 min from the baseline. There were significant increases in the heart rate (mean changes: 50 ± 13.3 beats/min), breathing rate (mean changes: 9.8 ± 4.1 breaths), local skin temperature (mean changes: 3.4 ± 1.9 °C), electrodermal activity at the right wrist (mean changes: 7.1 ± 3.8 µS/cm), and subjective physical fatigue (mean changes: 8.8 ± 0.6 levels) at the end of the simulated construction task (*p* < 0.05). Heart rate and breathing rate at 15 and 30 min were significantly correlated with the corresponding subjective Borg scores (*p* < 0.01). Local skin temperature at 30 min was significantly correlated with the corresponding Borg scores (*p* < 0.05). However, electrodermal activity at the right wrist was not associated with Borg scores at any time points. The results implied cardiorespiratory parameters and local skin temperature were good surrogates for measuring physical fatigue. Conversely, electrodermal activity at the right wrist was unrelated to physical fatigue. Future field studies should investigate the sensitivity of various cardiorespiratory and thermoregulatory parameters for real time physical fatigue monitoring in construction sites.

## 1. Introduction

The construction industry is known to have stressful work environments given the high physical demand of construction tasks and extreme working conditions [1,2,3]. As such, construction workers are at risk of experiencing physical strain and physical fatigue [4], which in turn may heighten the risk of musculoskeletal injuries in construction workers. Importantly, physical fatigue has more profound effects on older workers than younger workers because older adults have decreased physical working capacity, muscle mass, and cardiac output [5]. Given the aging construction workforce in many parts of the world, it has become the top priority of construction site managers and workers to lower the risk of work-related musculoskeletal disorders/injuries [6].

Construction workers usually experience physical fatigue because construction works typically involves physically demanding and repetitive tasks, manual labor, and often performs outdoor works in harsh and humid environmental conditions [7]. The presence of physical fatigue will increase the risk of human errors at workplaces [8,9], which may lead to construction accidents [10,11,12,13,14], occupational injuries [15], and fall accidents [16,17,18]. Therefore, continuous monitoring and prevention of physical fatigue is utmost important to ensure occupational health and safety of construction workers.

The development of new wearable technologies for real-time monitoring of physiological measures (e.g., heart rate, heart rate variability, skin temperature, breathing rate, electrodermal activity) have opened up opportunities for objective, uninterrupted, and continuous monitoring of physical fatigue during construction tasks. Under physical stress or load, the sympathetic nervous system will increase its activity to generate some physiological responses (e.g., increased heart rate (HR) and breathing rate (BR), increased skin temperature, and conductance). By measuring these responses, it may be possible to identify physical fatigue [19]. Yi et al., [20] and Aryal et al., [21] have used HR, and HR and local skin temperature, respectively, to measure physical fatigue in construction workers. More recently, Umer et al. [22] used several physiological measures (e.g., HR, skin temperature, and BR) to estimate physical fatigue using a machine learning algorithm. Further, a few studies have reported that BR is more strongly associated with physical exertion as compared to HR during exercises (e.g., intermittent or continuous), or under certain experimental induced fatigue conditions such as heat exposure, hypoxia, and glycogen depletion [23,24,25]. Therefore, BR has been suggested as one of the “neglected physiological measures” for physical fatigue assessment and is recommended to be used to assess physical exertion during physical tasks [26]. 

In addition to HR and BR, thermoregulatory measures such as local skin temperature and electrodermal activity may have the potential to evaluate fatigue during construction tasks. Previous studies have found strong correlations between thermoregulatory measures and fatigue development during cycling [27] and construction tasks [21]. Following vigorous physical activity, human core body temperature increases while the thermoregulatory system tries to control the body temperature [28,29]. Specifically, the skin assists heat transfer from the core body to the surrounding area by sweating [30]. Therefore, assessing the pattern of thermoregulatory changes at various body parts may help monitor physical fatigue during construction activities.

While using wearable technologies to monitor both cardiorespiratory and thermoregulatory parameters may help detect physical fatigue, there is a paucity of research to exploring the possibility of continuous monitoring of cardiorespiratory and thermoregulatory responses during construction tasks. Although the adoption of multiple cardiorespiratory and thermoregulatory measures is better than using a single physiological measure in detecting physical fatigue [21,22], it is important to consider the costs of using multiple wearable sensors, as well as their acceptance by construction workers. Therefore, a better understanding of relative responsiveness of different physiological measures for physical fatigue is very important. Unfortunately, it remains unclear which cardiorespiratory and thermoregulatory measures are more responsive to physical fatigue. 

Given the above, the current study aimed to determine which cardiorespiratory and thermoregulatory measure(s) more responsive to the onset of physical fatigue during a simulated construction task.

## 2. Methods

### 2.1. Participants

A convenient sample of 25 healthy male university students (mean age, 31.8 ± 1.8 years) from the faculty of construction recruited. People with any history of cardio and pulmonary diseases, neurological disorders, or musculoskeletal disorders were excluded. The study was conducted in accordance with the Declaration of Helsinki, and the protocol approved by the Ethical Committee of The Hong Kong Polytechnic University (Reference Number: HSEARS20190824004). Before data collection, the experimental procedures were explained to each participant and an informed consent was obtained. The demographics and physical health related data were collected using a customized self-reported questionnaire.

### 2.2. Simulated Fatigue Task

**Figure 1:**
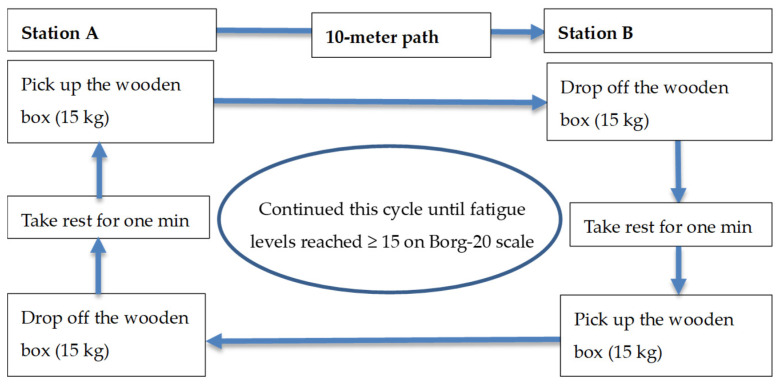
The design of the simulated experimental fatigue task.

The participants were requested to perform a simulated construction task until they reached self-determined exhaustion. The simulated construction task was manual material handling, which is the most common and physically demanding construction task. Participants were asked to carry a weight of 15 kg, which is a typical weight of material being handled on a construction site [31]. The temperature and relative humidity in the laboratory were set at 30 °C and 65%, respectively. The other environmental parameters such as solar radiation and wind speed were not controlled as the current study was conducted at a basement area where the sources of solar radiation and wind speed were very limited. The simulated manual material handling task was conducted using an experimental setup as described in previous research with some modifications (Figure 1) [21,32]. Notably, the experimental setup comprised a station A, a 10-m walking platform, and a station B. Each participant was instructed to pick up a 15 kg ergonomically-designed wooden box from station A, carry it along a path of 10 m, then drop it at station B, and take a one-minute rest prior to picking up the weight and carrying it to station A again. Participants were asked to continue this task cycle until they achieved a fatigue level > 15 out 20 on Borg-20 scale (perceiving hard exertion level). The Borg-20 scale is a ‘relative’ scale for rating perceived physical exertion. It ranges from 6 (no feeling of exertion) to 20 (very, very hard feeling of exertion). The cutoff score of 15 was chosen for fatigue as Borg suggested that if an exercise test (the simulated task in this study) aims to study physiological symptoms of fatigue, the task is often stopped at a HR between 150 and 170 beats/minute for individual aged between 30 and 50 years [33]. These HRs are corresponding to Borg scale ratings between 15 and 17.

### 2.3. Cardiorespiratory and Thermoregulatory Measurements

The cardiorespiratory (HR and BR) and thermoregulatory (skin temperature and electrodermal activity) measures were assessed at baseline, 15 min, and 30 min during fatigue task. Similarly, to gauge the subjective perception of physical fatigue, the Borg-20 scale was used at the respective times (e.g., baseline, 15 min, and 30 min). Additionally, the physiological stress index (PSI) was used to calculate the thermal strain experience by the participants. The following equation was used to calculate the PSI [34]:

$$PSI=\left[5\times \frac{Tpost-Tpre}{39.5-Tpre}+5\times \frac{HRpost-HRpre}{180-HRpre}\right];$$

where Tpre and Tpost, were local skin temperature measurements at baseline and after the fatigue task, respectively; HRpre and HRpost, were heart rate measurements at baseline and after the fatigue task, respectively. 

### 2.4. Equipment

**Figure 2:**
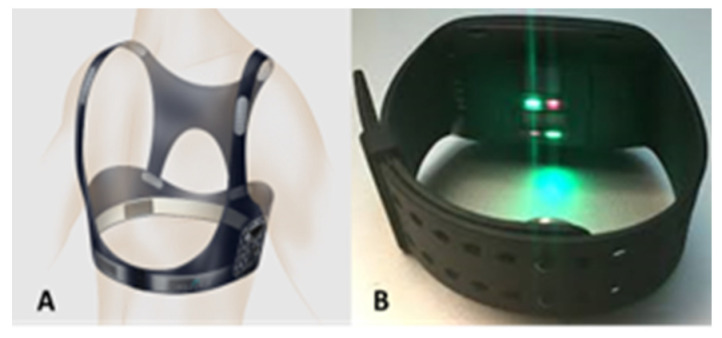
(**A**) Equivital LifeMonitor vest (**B**) E4 photoplethysmography (PPG) wristwatch (Picture reproduced with permission).

In order to obtain HR, BR, and local skin temperature, a new technology namely EQ02 LifeMonitor system (Equivital™, Cambridge, UK) (Figure 2A) was used. The EQ02 LifeMonitor system consists of a textile vest embedded with many sensors. It was designed to measure real-time electrocardiography (ECG) and local skin temperature. The vest was worn in a manner so that its sensors were positioned approximately over serratus anterior muscles (Figure 2A). The system has shown excellent reliably in measuring HR (ICC = 0.99; r = 0.98), BR (ICC = 0.96; r = 0.97), and local skin temperature (ICC = 0.97; r = 0.96) [35]. To assess electrodermal activity at the right wrist, a photoplethysmography (PPG) wristwatch (Empatica E4) (Figure 2B) was used. The PPG wristwatch is composed of six light emitting diodes and photoreceptors to measure HR based on the variation in skin blood flow at the right wrist. It also has two electrodes to measure the electrodermal activity of the skin at the right wrist. The reliability of the PPG wristwatch for measuring electrodermal activity was not tested earlier while performing certain physical tasks. To obtain a subjective benchmarking of physical fatigue, the Borg-20 scale was utilized [33]. It was a reliable and valid subjective tool to monitor fatigue development [36,37,38].

A pilot study (n = 10) was conducted to establish the test–retest reliability of using the EQ02 LifeMonitor system and a PPG wristwatch to monitor HR, BR, local skin temperature, and electrodermal activity during a simulated construction task. The equipment demonstrated an excellent test–retest reliability for measuring HR (ICC = 0.97) and a good test–retest reliability for measuring BR (ICC = 0.77), local skin temperature (ICC = 0.76), and electrodermal activity at the right wrist (ICC = 0.80).

### 2.5. Statistical Analysis

Statistical analyses were conducted using SPPS version 22 (IBM Inc., Chicago, IL, USA). Descriptive statistics (mean and standard error) were conducted. Separate repeated measure ANOVAs were used to see the changes in the cardiorespiratory and thermoregulatory measures at different time points during the simulated construction task. Post hoc analysis was done with the Bonferroni adjustment to examine the difference between the three time points (e.g., 0 min vs. 15 min, 15 min vs. 30 min, and 0 min vs. 30 min). Additionally, Pearson’s correlation coefficients were used to evaluate the associations between cardiorespiratory or thermoregulatory measures and the subjective measure of physical fatigue and PSI during/after the simulated construction task. The alpha level was set at 0.05 for all the tests.

## 3. Results

Table 1 details the descriptive statistics of all the variables. Mean HR increased from 70.2 beats/min at baseline to 120.2 beats/min at the end of the simulated construction task. Similarly, mean BR increased from 17.9/min at baseline to 27.8/min at the end of the simulated construction task. Mean increases in the local skin temperature and electrodermal activity during the simulated construction task were 3.4 °C and 7.1 µS/cm, respectively. The mean increase in the subjective physical fatigue score was 8.8 levels during the simulated construction task. The calculated PSI indicated that the participants experienced nearly moderate levels of thermal stress during the simulated construction task. 

Table 2 indicates the cardiorespiratory and thermoregulatory responses during the simulated construction task. The ANOVA results revealed significant changes in HR, BR, local skin temperature, electrodermal activity, and subjective physical fatigue scores (all *p* < 0.05). The increases in HR, BR, and local skin temperature were rapid and large in first 15-min task. The increments of these measures in the subsequent 15 min were significantly smaller than the first 15 min. However, the increase in the electrodermal activity was almost double at the end of the 30-min task as compared to the first 15 min (4.1 versus 2.9). 

Table 3 presents the correlations between cardiorespiratory or thermoregulatory measures and subjective physical fatigue or PSI scores. Notably, HR and BR at 15 and 30 min were significantly correlated with the respective subjective physical fatigue scores (*p* < 0.01). Local skin temperature at 30 min was significantly correlated with the corresponding subjective physical fatigue scores (*p* < 0.05). However, the electrodermal activity was not associated with the subjective physical fatigue scores at any time points. Both objective (e.g., cardiorespiratory and thermoregulatory) and subjective (e.g., Borg score) fatigue scores were significantly correlated with the thermal stress (e.g., PSI) experienced by the participants during 30 min of the simulated construction task.

**Figure 3:**
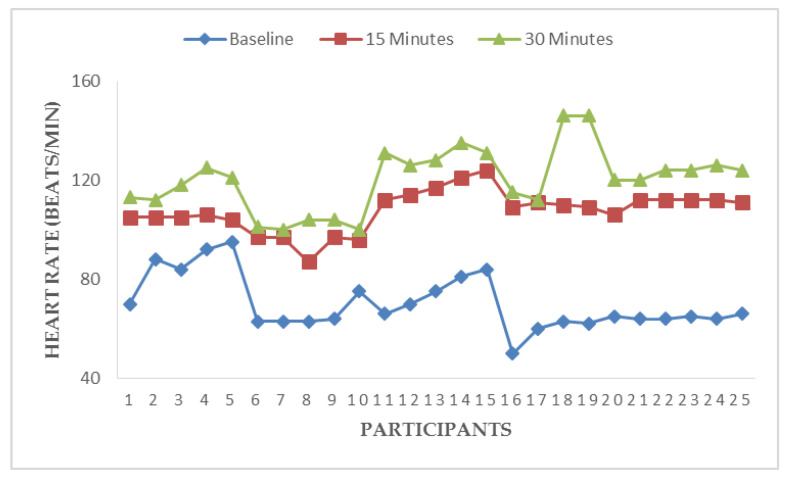
Responses of heart rate during a simulated construction task.

**Figure 4:**
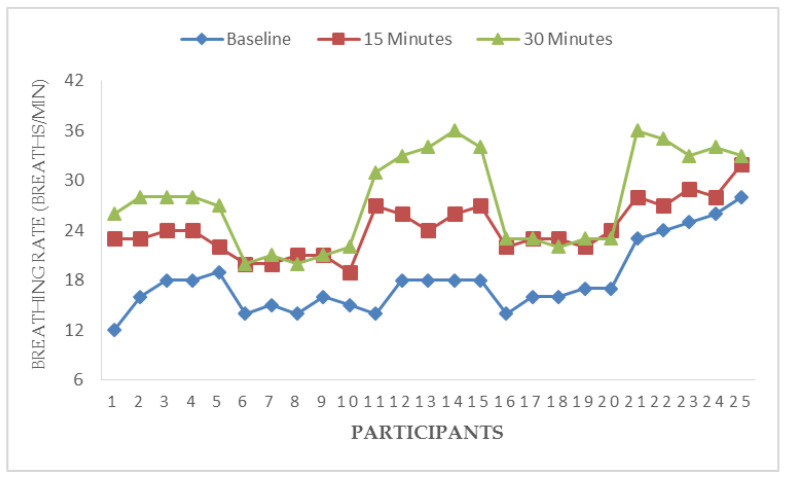
Responses of breathing rate during a simulated construction task.

**Figure 5:**
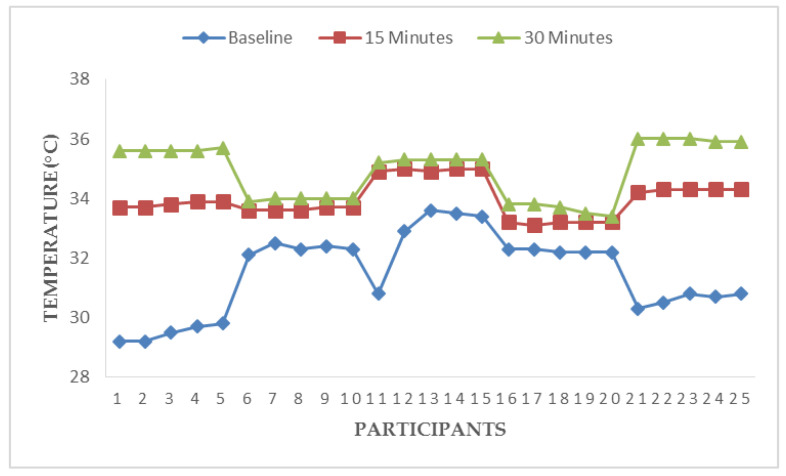
Responses of local skin temperature during a simulated construction task.

**Figure 6:**
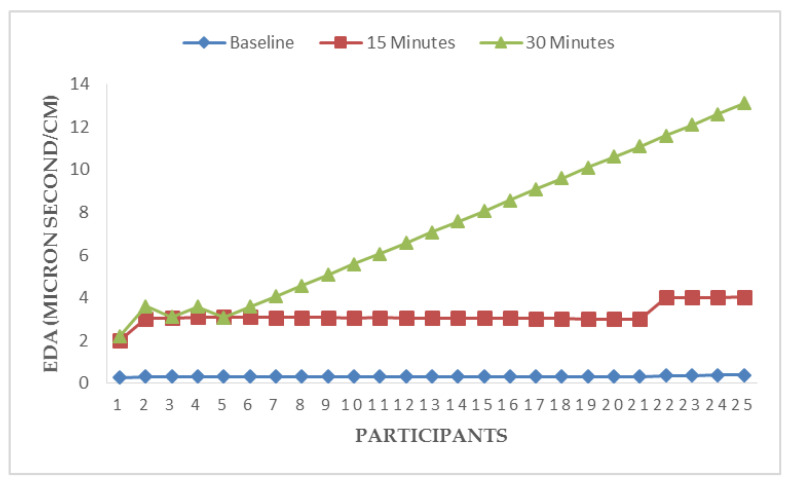
Responses of electrodermal activity (EDA) at the right wrist during a simulated construction task.

Figure 3, Figure 4, Figure 5 and Figure 6 illustrate responses of cardiorespiratory and thermoregulatory measures during the simulated task in all the participants. Participants had a minimum HR of 50 beats/min (Participant no. 16) and a maximum of 95 beats/min (Participant no. 5) at baseline. However, two participants had a minimum HR of 100 beats/min and a maximum of 146 beats/min at the end of the task (at 30 min). Additionally, participants had a minimum BR of 12 breaths/min (Participant no. 1) and a maximum of 28 breaths/min at baseline (Participant no. 5). Similarly, two participants had a minimum BR of 20 breaths/min and a maximum of 36 breaths/min at the end of task (at 30 min). Furthermore, two participants had a minimum local skin temperature of 29.2 °C and one participant had a maximum of 33.6 °C at baseline. However, one participant had a minimum local skin temperature of 33.4 °C and three participants had a maximum temperature of 36 °C at 30 min. Moreover, one participant had a minimum electrodermal activity of 0.27 µS/cm and two participants had a maximum electrodermal activity of 0.37 µS/cm at baseline. At the end of 30 min, one participant had a minimum electrodermal activity of 2.2 µS/cm and a maximum of 13.1 µS/cm.

## 4. Discussion

This is the first study to simultaneously compare various cardiorespiratory (HR and BR) and thermoregulatory (local skin temperature and electrodermal activity) parameters and physical fatigue following a simulated construction task. These results substantiated that HR, BR, and local skin temperature were potential good indicators for monitoring physical fatigue during or at the end of the simulated task. However, electrodermal activity was unrelated to the subjective physical fatigue score during the simulated task. Interestingly, both measured cardiorespiratory and thermoregulatory parameters were significantly related to PSI.

The strong association between HR and Borg-20 scores in the current study corroborates previous findings that HR can be used as a physiological measure for detecting as an early sign of physical stress on the body [39,40]. Previous studies indicated that the normal range of safe HR during heavy activities was between 120 and 160 beats/min [39,41]. Our participants’ HR at the end of the 30-min task was between 100 and 146 beats/min, and the average post-task increase in HR was 50 beats/min (70% increments from baseline). Such HR increments differed from previous research, which reported approximately 38% elevation of the working HR as compared to the resting HR [42]. However, findings from the two studies should not be directly compared because participants in the previous study were cabin field machine operators who were involved in less strenuous tasks than the materials handling tasks. In addition, since our participants were healthy university students, it was possible that they were not well adapted to the manual material handling task. 

Since HR directly affects the blood supply to muscles, greater physical workloads are associated with higher HR. Previous study reported a strong correlation between HR and Borg scores (r = 0.74) during incremental exercise tests on cycle ergometers or treadmills [43]. Scherr et al. [44] reported a moderate association between HR and Borg scores (r = 0.62) in healthy individuals. However, poor correlation between the two variables has also been reported. Chen et al. [45,46] reported a poor correlation between HR and Borg score (r = 0.27) during static and partial dynamic exercise. Mehta et al. [47] also reported poor correlation between subjective and objective measures (r = 0.25) of fatigue in the oil and gas extraction workers. However, these studies were limited by their small sample sizes (only 12 [46] and 10 [47] participants). Additionally, Mehta et al. [47] used the occupational fatigue exhaustion/recovery scale and fatigue assessment scale to measure subjective fatigue outcomes. These subjective fatigue scales are used to measure both physical and mental fatigue [48,49]. 

Our results showed that BR had the second strongest relationship with the Borg-20 score throughout the task. Although previous construction studies did not directly used BR to measure physical fatigue, BR has been recently regarded as a “neglected physiological measure” for fatigue assessment during physical tasks [26]. As such, the BR measure may be used to assess physical fatigue during construction tasks [26]. A few studies have reported that BR measure is more strongly associated with physical strain as compared to other physiological measures such as oxygen consumption and HR during various intermittent or continuous exercises, and under experimentally induced fatigue conditions (e.g., heat exposure, hypoxia, and glycogen depletion) [23,24,25,26]. The strong association between physical exertion and BR measure may be attributed to the fact that the central nervous system controls the BR in order to provide sufficient oxygen to support the oxidative phosphorylation of adenosine triphosphate during aerobic muscle contraction. Therefore, BR is positively related to the intensity of motor effort during physical activity [23,26]. However, this relation may not persist when muscle cells rely on anaerobic metabolism to generate energy during muscle fatigue. 

In the present study, the local skin temperature was increased at the end of the simulated task but not during the task. The increased local skin temperature alone may not be able to detect physical fatigue during the task. Aryal et al. [21] used local skin temperature and HR measures together to develop a fatigue assessment model that showed 72% prediction accuracy for identifying fatigue using both skin temperature and HR data. Similarly, Jebelli et al. [19] confirmed the ability of combined physiological measures including local skin temperature in detecting physical workloads during different construction tasks. Given that local skin temperature can be affected by ventilation and relative humidity in the atmosphere, as well as the placement of the sensors in the body locations, combining local skin temperature with HR and/or BR [21,22] may provide better monitoring/prediction of physical fatigue in construction workers. Additionally, future studies should consider skin temperature at multiple body parts to get a more accurate result.

Our results suggest that the electrodermal activity at the wrist during construction tasks could not measure physical fatigue. Although prior research has found electrodermal activity as a valuable biomarker for assessing stress-related effects on human body [50,51], the increase in electrodermal activity was mainly related to mental stress [52,53]. Interestingly, the current study failed to find a significant correlation between subjective fatigue scores (Borg-20) and the electrodermal activity at the wrist during the simulated construction task. Since ventilation, surrounding temperature, and hydration of a person may affect the electrodermal activity of skin, our findings should be interpreted with caution. Given the strong correlation between electrodermal activity at the right wrist and PSI, refined measurements of electrodermal activity may be needed to measure physical fatigue. Future studies should consider placing sensors at multiple body parts (e.g., thighs, back, chest) instead of placing at the right wrist alone to verify the relation between electrodermal activity and physical fatigue.

The current study had some limitations. First, the sample size was small, however, large differences in HR and BR measures during the simulated construction task suggested that these physiological measures were useful indicators for measuring physical fatigue during construction tasks. Future research should validate the findings in a large sample of construction workers in the field. Second, instead of using a subjective fatigue scale, more objective measures of physical fatigue such as blood lactate levels can be used in future studies to estimate the actual relationship between physiological measures and physical fatigue. Third, our participants followed a strict protocol rather than a self-paced protocol to perform the 30-min task in a laboratory, which did not truly reflect the actual occupational settings during a full 8-h work-shift. Since workers usually spend a significant amount of time on irregular work breaks to dissipate heat or to recover from fatigue [54,55,56], future studies should investigate whether this approach can prevent workers from physical fatigue during a typical work-shift. Fourth, although the current study used high temperature and humidity to simulate a harsh working environment, other environmental factors (e.g., solar radiation, and wind speed) were not considered. Further, unlike our experiment, the temperature and relative humidity in real workplaces may fluctuate throughout the day. Future field research should determine the best physiological measure(s) for continuous monitoring of physical fatigue among construction workers during a typical work-shift. 

## 5. Conclusions

The temporal changes in the cardiorespiratory and thermoregulatory measures during the simulated construction task, as well as their significant relations with self-perceived fatigue indicate that the cardiorespiratory and thermoregulatory measures (except electrodermal activity at the right wrist) are good surrogates for assessing physical fatigue. Future field studies should investigate the sensitivity and accuracy of using a single or a combination of these cardiorespiratory and thermoregulatory measures to monitor physical fatigue among construction workers. 

## Figures and Tables

**Table 1:** Descriptive Statistics of the participants.

Variables	Mean	Std. Error	95% CI
Age (Y)	31.8	1.8	26.7–36.8
Height (m)	1.6	0.03	1.6–1.7
Weight (kg)	67.6	1.1	64.5–70.7
Heart rate at 0 min, Beats/min	70.2	2.2	65.6–74.8
Heart rate at 15 min, Beats/min	107.6	1.7	104.2–111.1
Heart rate at 30 min, Beats/min	120.2	2.6	115.1–125.5
Breathing rate at 0 min, N	17.9	0.83	16.3–19.7
Breathing rate at 15 min, N	24.2	0.64	22.9–25.5
Breathing rate at 30 min, N	27.8	1.13	25.4–30.1
Local skin temperature at 0 min, °C	31.5	0.28	30.9–32.1
Local skin temperature at 15 min, °C	33.9	0.12	33.7–34.2
Local skin temperature at 30 min, °C	34.9	0.19	34.5–35.3
Electrodermal activity at 0 min, µS/cm	0.32	0.01	0.31–0.33
Electrodermal activity at 15 min, µS/cm	3.17	0.09	2.9–3.4
Electrodermal activity at 30 min, µS/cm	7.3	0.68	5.9–8.7
Subjective fatigue score (BORG - 20) at 0 min	6.6	0.17	6.3–6.9
Subjective fatigue score (BORG - 20) at 15 min	13.1	0.17	12.8–13.5
Subjective fatigue score (BORG - 20) at 30 min	15.4	0.20	15.0–15.8
Physiological stress index	4.13	0.99 *	3.74–4.52

* Standard deviation.

**Table 2:** Cardiorespiratory and thermoregulatory measures during a simulated construction task.

Variables	Measurement Times, Mean (SD)			ANOVA		Post Hoc Analysis (Bonferroni)		
	0 min	15 min	30 min	F	P	0 min vs. 15 min	15 min vs. 30 min	0 min vs. 30 min
Heart Rate, Beats/min	70.2 (11.1)	107.6 (8.3)	120.2 (12.7)	202.27	0.001 *	d = −37.4	d = −12.6 (0.001 *)	d = −50.0
						(*p* = 0.001 *)		(*p* = 0.001 *)
Breathing rate, N	17.9 (4.1)	24.2 (3.2)	27.8 (5.6)	104.38	0.001 *	d = −6.2	d = −3.6 (0.001 *)	d = −9.8
						(*p* = 0.001 *)		(*p* = 0.001 *)
Local skin temperature, °C	31.5 (1.4)	33.9 (0.62)	34.9 (0.94)	68.38	0.001 *	d = −2.5	d = −0.92 (0.001 *)	d = −3.4
						(*p* = 0.001 *)		(p = 0.001 *)
Electrodermal activity, µS/cm	0.32 (.02)	3.17 (0.44)	7.3 (3.4)	85.87	0.001 *	d = −2.9	d = −4.1 (0.001 *)	d = −7.1
						(*p* = 0.001 *)		(*p* = 0.001 *)
Subjective fatigue (Borg, 6–20)	6.6 (0.86)	13.1 (0.83)	15.4 (1.0)	816.84	0.001 *	d = −6.5	d = −2.3 (0.001 *)	d = −8.8
						(*p* = 0.001 *)		(*p* = 0.001 *)

* Data are statistically significant at the *p* < 0.05 level (2-tailed); d = Mean Difference.

**Table 3:** Correlations of cardiorespiratory and thermoregulatory measures with subjective fatigue scores.

Pearson Correlation Test	Borg, 0 min	Borg, 15 min	Borg, 30 min	Physiological Stress Index
Heart rate at 0 min	0.931 *			
Heart rate at 15 min		0.696 *		
Heart rate at 30 min			0.821 *	0.411 *
Breathing rate at 0 min	0.121			
Breathing rate at 15 min		0.600 *		
Breathing rate at 30 min			0.701 *	0.739 *
Local skin temperature at 0 min	0.204			
Local skin temperature at 15 min		0.071		
Local skin temperature at 30 min			0.465 *	0.727 *
Electrodermal activity at 0 min	−0.367			
Electrodermal activity at 15 min		0.259		
Electrodermal activity at 30 min			0.369	0.836 *
Physiological stress index			0.981 *	

* Data are statistically significant at the *p* < 0.01 level (2-tailed).
